# Stochastic modeling and parameter estimation of turbogenerator unit of a thermal power plant under classical and Bayesian inferential framework

**DOI:** 10.1371/journal.pone.0292154

**Published:** 2023-10-20

**Authors:** Ashish Kumar, Ravi Chaudhary, Kapil Kumar, Monika Saini, Dinesh Kumar Saini, Punit Gupta

**Affiliations:** 1 Department of Mathematics & Statistics, Manipal University Jaipur, Jaipur, India; 2 Department of Statistics, Central University of Haryana, Mahendragarh, India; 3 Department of Computer & Communication Engineering, Manipal University Jaipur, Jaipur, India; 4 School of Computer Science, University College Dublin, Ireland; Chitkara University, INDIA

## Abstract

The work reported in present study deals with the development of a novel stochastic model and estimation of parameters to assess reliability characteristics for a turbogenerator unit of thermal power plant under classical and Bayesian frameworks. Turbogenerator unit consists of five components namely turbine lubrication, turbine governing, generator oil system, generator gas system and generator excitation system. The concepts of cold standby redundancy and Weibull distributed random variables are used in development of stochastic model. The shape parameter for all the random variables is same while scale parameter is different. Regenerative point technique and semi-Markov approach are used for evaluation of reliability characteristics. Sufficient repair facility always remains available in plant as well as repair done by the repairman is considered perfect. As the life testing experiments are time consuming, so to highlight the importance of proposed model Monte Carlo simulation study is carried out. A comparative analysis is done between true, classical and Bayesian results of MTSF, availability and profit function.

## 1. Introduction

The increasing demand and technological advancements inclined the complexity of industrial and mechanical systems. The products generated by these industries are extensively used in day-to-day life of human being. The thermal power plant is also a such system which is prominently contribute to energy generation sector in most of the countries. Availability, mean time to system failure and performance of the thermal power plants attract the attention of system designers to assess the effectiveness of plants during last few decades. Various subsystems of these plants critically influence the performance. Turbogenerator is a prominent component of thermal power plant that influences the performance of whole plant. So, reliability characteristics evolution of these components become necessary to evaluate performance of the plant. Several methodologies like fault tree analysis, failure mode effect analysis, Markovian approach and reliability block diagram approach are used in previous studies under various kind of failure distributions. Such a distribution to investigate reliability of industrial systems is proposed by Weibull [1]. Weibull distribution have wide applicability in life testing, reliability modeling and estimation due to its flexible shapes of the failure rate functions.

Provision of spare component is also a reliability enhancement technique that can be used in such systems. Masters et al. [2] developed a model for confidence interval estimation of availability function for Weibull distributed operating system. Dhillon and Anuda [3] developed a stochastic model under arbitrary failure rates and common cause failures. Coit [4] optimized the redundancy of components in non-repairable systems. Yadavalli et al. [5] used concept of preparation time to develop asymptotic confidence limits for availability function of parallel systems. Lim et al. [6] developed bootstrap confidence interval for steady state availability of systems. Yadavalli et al. [7] conducted a Bayesian study for two-unit system under impact of common cause shock failures. Chien et al. [8] developed asymptotic confidence limits for a repairable system having imperfect service facility. Ke et al. [9] performed the Bayesian estimation of standby system under imperfect coverage. Hsu et al. [10] done Bayesian and asymptotic estimation under reboot and imperfect coverage for repairable system. Gupta et al. [11] done the cost analysis of non-identical unit’s system considering Weibull distribution for failure and repair rates. Singh et al. [12] drawn some statistical inferences for a time dependent dynamical system. Chaturvedi et al. [13] developed a robust model for Weibull distribution under Bayesian framework.

Kishan and Jain [14] conducted the parameter estimation for a parallel unit system to evaluate the reliability measures. It is considered that all time dependent random variables are Weibull distributed having common shape parameter. Kumar and Saini [14] proposed a stochastic model for single unit system to assess the impact of preventive maintenance under Weibull distribution. Liu et al. [15]conducted the reliability evaluation of a system of non-identical units under fuzzy environment. Kumar et al. [16] studied the effect of hot and cold standby redundancy on availability of thermal power plants. Kumar and Garg [17] estimated parameters of generalized inverted Rayleigh distribution under random censoring. Pariaman et al. [18] discussed several methodologies for availability enhancement of thermal power plants. Dongliang et al. [19] used phase time distribution for reliability estimation of non-identical unit systems. Kumar et al. [20–22] investigated the impact of various kind of priorities and preventive maintenance on systems of Weibull distributed random variables. Chopra and Ram [23] proposed a stochastic model for parallel system with waiting time. Dey et al. [24] provided an extension of generalized exponential distribution having application in Ozone data. Gupta and Singh [25] conducted classical and Bayesian analysis of Weibull distribution under outliers. Han et al. [26] explored the needs of Bayesian statistics in various studies.

Pundir et al. [27] developed a stochastic framework for parallel system of non-identical units having priority in repair disciplines. Kumar and Kadyan [28, 29] proposed reliability models for performance evaluation of industrial system using supplementary variable technique. Kumar and Kumar [30] estimate various statistical properties of inverse Weibull distribution under random censoring. Saini and Kumar [31] developed a stochastic model for single unit system under abnormal environmental conditions to assess impact of inspection and degradation. Saini et al. [32] proposed a stochastic model to evaluate the profit of redundant system under priority. Pundir et al. [33] analysed the impact of presence of prior on reliability estimation of standby system. Patawa et al. [34] drawn various inferences for reliability measures of non-identical system with standby redundancy and waiting time in Bayesian framework. Rathi et al. [35] developed a model for reliability improvement using redundancies and Markov process.

Though, a lot of work has been carried out in the direction of reliability evaluation of industrial system, but it is focused only on modelling, MTSF, steady state availability and performance evaluation by considering constant failure and repair rates of components. The estimation of the parameters is still not extensively explored for industrial system specially in field of thermal power plants. The reliability modelling and classical & Bayesian estimation of reliability measures of turbogenerator unit yet not discussed in literature so far. So, in the present work a novel stochastic model for turbogenerator system comprises with five components of thermal power plant is proposed by considering Weibull distribution for failure and repair rates having different scale parameter and common shape parameter. As Weibull distribution is the most popular in reliability modeling and estimation due to its flexible shapes of the failure rate functions. To extract concrete findings from stochastic model simulation study is conducted. The following system reliability measures, which are useful for plant designers and maintenance managers, are derived using semi-Markovian approach and regenerative point technique:

- Steady state transition probabilities associated with various states of turbogenerator system
- Mean sojourn times associated with various regenerative states of turbogenerator system
- True and estimated values of mean time to system failure (MTSF)of turbogenerator system
- True and estimated values of steady state availability of turbogenerator system
- True and estimated values of profit of turbogenerator system

Due to random behaviour of lifetime of the components of turbogenerator the parameter of associated distribution is estimated in classical and Bayesian framework. The posterior densities are not easy to simulate directly so Metropolis-Hastings algorithm of the MCMC procedure is utilized to generate the random samples from this posterior density. The Monte Carlo simulation technique is employed to derive the numerical values of reliability measures in classical and Bayesian framework. The mean square error (MSE), confidence interval length along with MTSF, availability and profit are evaluated in classical framework while under Bayesian framework posterior mean square error, width of highest posterior density are computed. To highlight the importance of study, a comparative analysis is also made through numerical results and graphs. The whole manuscript is organized into five sections including the current introduction section. Section 2 includes the notations and system description. Tall the reliability measures obtained in section 3 while section 4 devoted to the estimation of parameters in classical and Bayesian framework. Concluding remarks are made in section 5.

## 2. Notations and system description

In this section the system description of turbogenerator and notation used for model development are appended.

### 2.1 Notations

S_i_: *i*^*th*^ state of the turbogenerator

**θ**_**i**_**/β**_**i**_ (i = 1, 2, 3, 4, 5): Scale parameter of failure/repair time distribution for *i*^*th*^ unit

**η:** Shape parameter of failure/repair time distribution of each unit

***f***_***i***_**(t)**: Failure rate of *i*^*th*^ unit where $f_{i}(t)=\theta_{i}\eta t^{\eta -1}e^{-\theta_{i}t^{\eta }},\theta_{i}>0,t>0$

***g***_***i***_**(t)**: Repair rate of *i*^*th*^ unit where $g_{i}(t)=\beta_{i}\eta t^{\eta -1}e^{-\beta_{i}t^{\eta }},\beta_{i}>0,t>0$

***q***_***ij***_**(*t*)/*Q***_***ij***_**(*t*):** Pdf and c.d.f. of one step or direct transition time from S_i_ ∈ E to S_j_ ∈ E

***p***_***ij***_**(*t*):** Steady state transition probability from state S_i_ to S_j_ such that, $p_{ij}=\underset{t\to \infty }{\lim}Q_{ij}(t)$

$p_{ij}^{\left(k\right)}(t)$: steady state transition probability from state S_i_ to S_j_ via S_k_ such that, $p_{ij}^{\left(k\right)}=\underset{t\to \infty }{\lim}Q_{ij}^{\left(k\right)}(t)$

**Z**_**i**_ **(t):** Probability that system sojourns in state S_i_ up to time t

*μ*_*i*_: Mean sojourn time in state Si i.e., $\mu_{i}=\int_{0}^{\infty }P(T_{i}>t)$

***R***_***i***_**(*t*):** Reliability of the system at time t when system starts from S_i_ ∈ E

***A***_***i***_**(*t*):** Probability that the system will be operative in state S_i_ ∈ E at epoch t

***B***_***i***_**(*t*):** Probability that the repairman will be busy in state S_i_ ∈ E at epoch t

***P***_***i***_**(*t*):** Profit incurred by the system during interval (0, t)

**: Symbol for Laplace Transform of a function i.e., $Q_{ij}^{**}(s)=\int_{0}^{\infty }q_{ij}(t)e^{-st}dt$

•: Regenerative point

X: Non-regenerative point

*A*_*o*_: Turbine governing unit (A) is operative

*B*_*o*_: Turbine lubrication unit (B) is operative

*C*_*o*_: generator oil system (C) is operative

*D*_*o*_: generator gas system (D) is operative

E_o_: generator extinction system € is in normal mode and operative

E_s_ Unit-E is in standby mode

*a*_*r*_/*a*_*wr*_: Turbine governing unit (A) is either in repair/waiting for repair

*b*_*r*_/*b*_*wr*_: Turbine lubrication unit (B) is either in repair/waiting for repair

*c*_*r*_/*c*_*wr*_: generator oil system (C) is either in repair/waiting for repair

*d*_*r*_/*d*_*wr*_: generator gas system (D) is either in repair/waiting for repair

e_wr_: Unit-E is in non-operative mode and under waiting for repair

e_r_: Unit-E is in non-operative mode and under repair

### 2.2 System description

**Fig 1:**
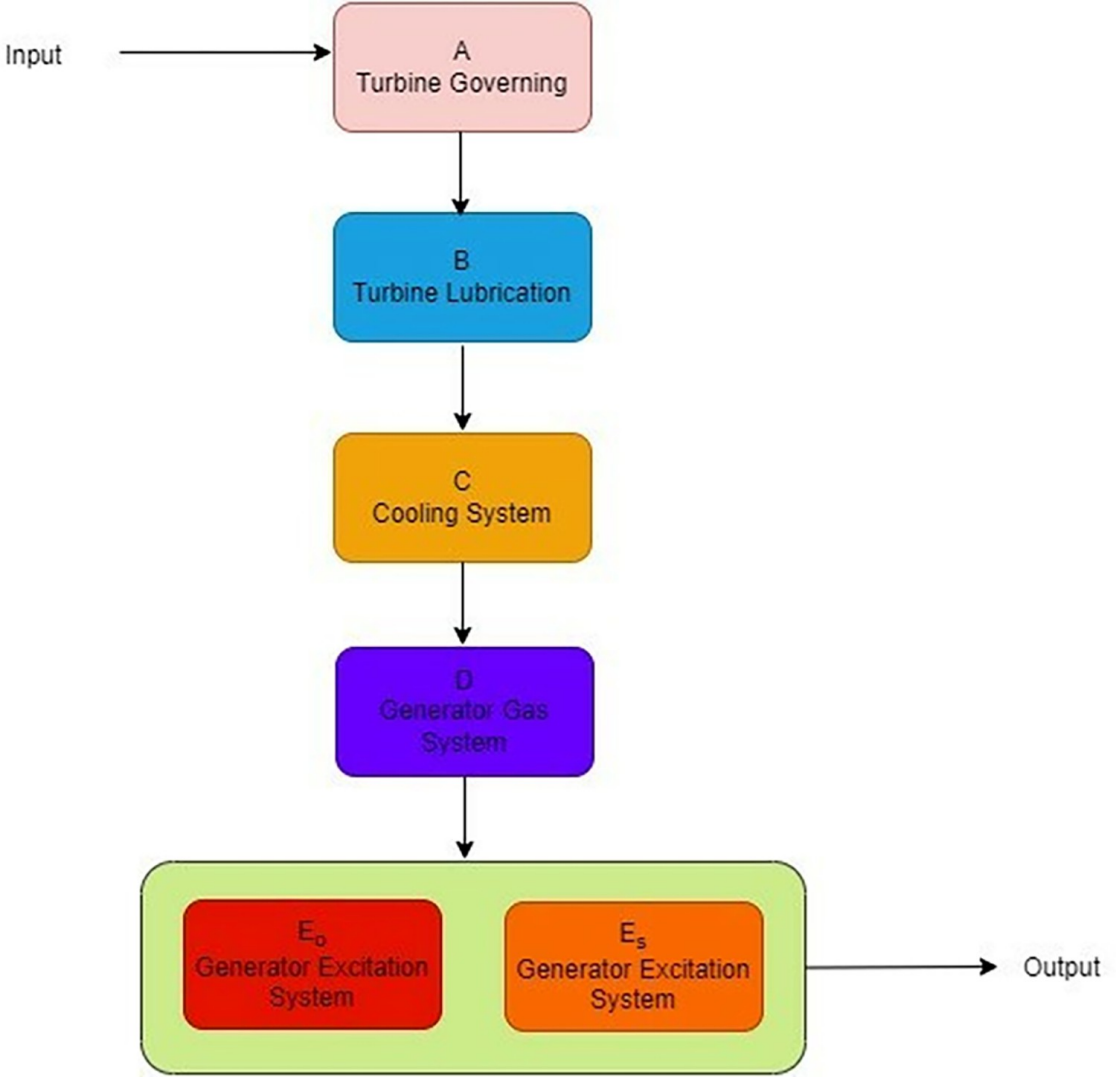
Flowchart of turbogenerator system.

**Fig 2:**
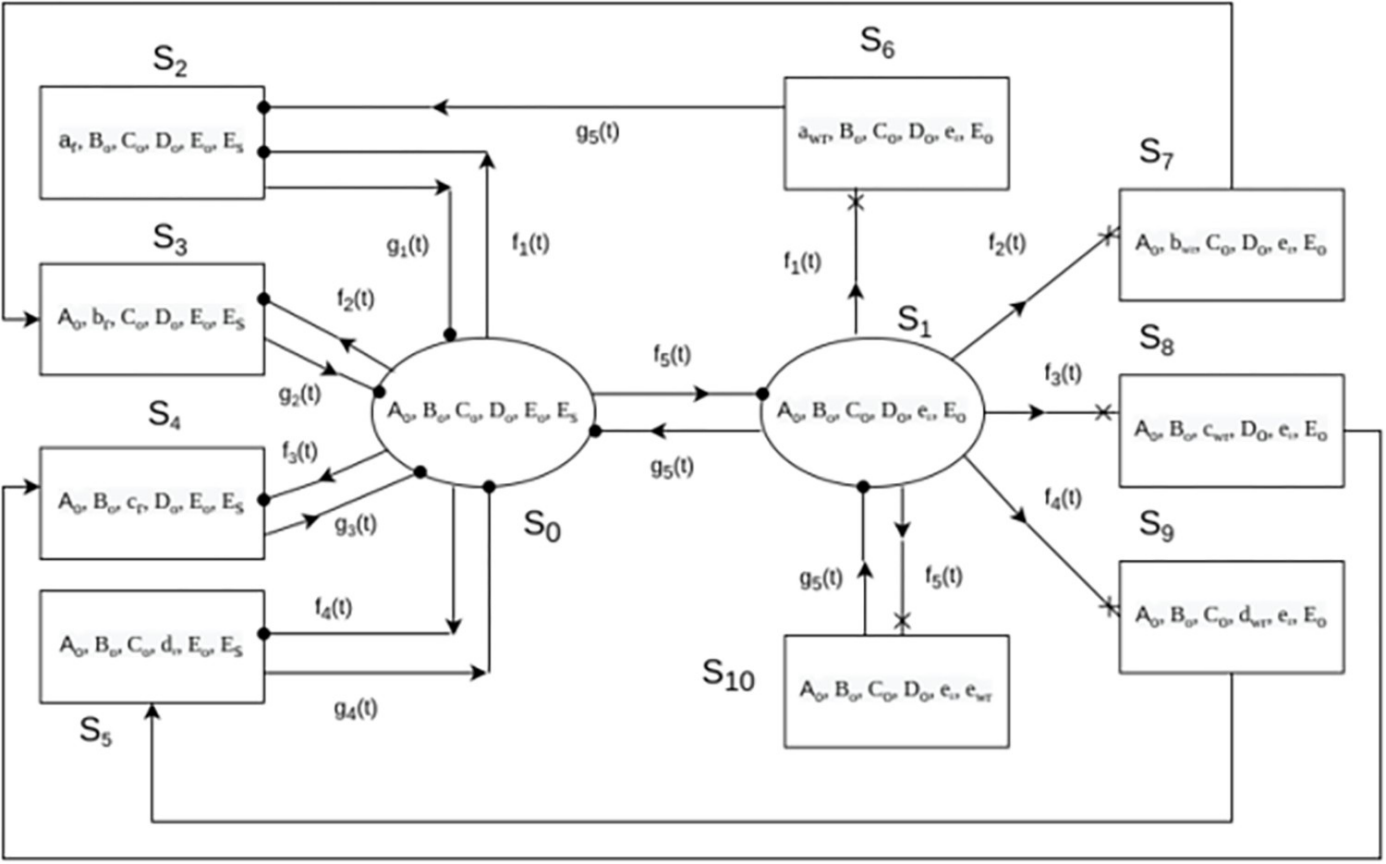
State transition diagram of turbogenerator system.

The turbogenerator is a critical component of thermal power plant and its availability influence the performance of whole plant in production of electricity. The considered turbogenerator [36] in present study is installed in a thermal power plant in India that produce 500 MW electricity. It consists of five subsystems (i) turbine governing “A” (ii) turbine lubrication “B”, (iii) generator oil system “C” (iv) generator gas system “D” and (v) generator extinction system “E”. There is no provision of standby component for turbine governing, turbine lubrication, generator oil system, and generator gas system while provision of one cold standby component is made for generator extinction system. The failure of single unit subsystems immediately resulted as the complete system failure. The flow chart of system is shown in Fig 1. The system works under a set of assumptions like failure and repair rates are statistically independent to each other, no multiple failures, standby units worked in full capacity and after repair unit worked as new one. Under this assumption, here the reliability characteristics of turbogenerator is assessed using regenerative point technique and semi-Markovian approach. A stochastic model is proposed and expressions for various reliability measures are derived. The failure and repair rates are obtained from time to failure and time to repair data. Further, the parameter estimation is done under classical and Bayesian inferential frameworks. The state transition diagram of the proposed stochastic model is shown in Fig 2.

## 3. Reliability measures of turbogenerator system

### 3.1 Transition probabilities

The state space of the turbogenerator system is discrete in nature having states {S_0_, S_1_, S_2_, S_3_, S_4_, S_5_, S_6_, S_7_, S_8_, S_9_, S_10_}. The probability of movement among these states is known as transition probability. Here, *p*_*ij*_ represent the transition from state ‘i’ to ‘j’. By simple probabilistic considerations value of *p*_*ij*_ is obtained by following expression for the non-zero elements of transition probability matrix (TPM):

$$p_{ij}=\underset{t\to \infty }{\lim}Q_{ij}(t)=\int_{0}^{\infty }q_{ij}(t)dt$$ (1)

The associated transition probability matric of present system is defined as:

$$X=\left[\begin{array}{ccc} p_{00} & \cdots & p_{0,10}\\ \vdots & \ddots & \vdots \\ p_{10,0} & \cdots & p_{10,10} \end{array}\right]$$

So, Eq (1) gives the values of all the entries of TPM (X) as the probability of transition from state S_0_ to state S_1_ with transition rate *f*_5_(*t*) without any transition to other states. The detailed procedure is presented in [1]. Mathematically,

$$Q_{01}(t)=\int_{0}^{\infty }f_{5}(t)\bar{F_{1}(t)}\bar{F_{2}(t)}\bar{F_{3}(t)}\bar{F_{4}(t)}dt$$

taking Laplace transform from both side

$$Q_{01}^{**}(s)=\int_{0}^{\infty }\theta_{5}\eta t^{\eta -1}e^{-(\theta_{1}+\theta_{2}+\theta_{3}+\theta_{4}+\theta_{5}+s)t^{\eta }}dt=\underset{s\to 0}{\lim}\frac{\theta_{5}}{\left(\theta_{1}+\theta_{2}+\theta_{3}+\theta_{4}+\theta_{5}+s\right)}$$

$${\Rightarrow p}_{01}=\underset{s\to 0}{\lim}Q_{01}^{**}(s)=\frac{\theta_{5}}{\left(\theta_{1}+\theta_{2}+\theta_{3}+\theta_{4}+\theta_{5}\right)}.$$

$$Similarly,p_{02}=\frac{\theta_{1}}{\left(\theta_{1}+\theta_{2}+\theta_{3}+\theta_{4}+\theta_{5}\right)},p_{03}=\frac{\theta_{2}}{\left(\theta_{1}+\theta_{2}+\theta_{3}+\theta_{4}+\theta_{5}\right)},p_{04}=\frac{\theta_{3}}{\left(\theta_{1}+\theta_{2}+\theta_{3}+\theta_{4}+\theta_{5}\right)},$$

$$p_{05}=\frac{\theta_{4}}{\left(\theta_{1}+\theta_{2}+\theta_{3}+\theta_{4}+\theta_{5}\right)},p_{16}=\frac{\theta_{1}}{\left(\theta_{1}+\theta_{2}+\theta_{3}+\theta_{4}+\theta_{5}+\beta_{5}\right)},p_{17}=\frac{\theta_{2}}{\left(\theta_{1}+\theta_{2}+\theta_{3}+\theta_{4}+\theta_{5}+\beta_{5}\right)},$$

$$p_{18}=\frac{\theta_{3}}{\left(\theta_{1}+\theta_{2}+\theta_{3}+\theta_{4}+\theta_{5}+\beta_{5}\right)},p_{19}=\frac{\theta_{4}}{\left(\theta_{1}+\theta_{2}+\theta_{3}+\theta_{4}+\theta_{5}+\beta_{5}\right)},p_{1,10}=\frac{\theta_{5}}{\left(\theta_{1}+\theta_{2}+\theta_{3}+\theta_{4}+\theta_{5}+\beta_{5}\right)}{,p}_{10}=\frac{\beta_{5}}{\left(\theta_{1}+\theta_{2}+\theta_{3}+\theta_{4}+\theta_{5}+\beta_{5}\right)},p_{20}=\underset{s\to 0}{\lim}Q_{20}^{**}(s)=1,p_{30}=\underset{s\to 0}{\lim}Q_{30}^{**}(s)=1,p_{40}=\underset{s\to 0}{\lim}Q_{40}^{**}(s)=1,p_{50}=\underset{s\to 0}{\lim}Q_{50}^{**}(s)=1,p_{62}=\underset{s\to 0}{\lim}Q_{62}^{**}(s)=1,p_{73}=\underset{s\to 0}{\lim}Q_{73}^{**}(s)=1,p_{84}=\underset{s\to 0}{\lim}Q_{84}^{**}(s)=1,p_{95}=\underset{s\to 0}{\lim}Q_{95}^{**}(s)=1,p_{10,1}=\underset{s\to 0}{\lim}Q_{10,1}^{**}(s)=1.$$

It is easily verified that sum of all entries of each row is unity.

### 3.2 Mean sojourn times

The average time spent by a system is known as mean sojourn time. The detailed procedure is presented in [1]. If *T*_*i*_ represent the average sojourn/survival time of turbogenerator at a particular state *S*_*i*_, then the mean sojourn time in the state *S*_*i*_ is evaluated using mathematical expressions:

$$\mu_{i}=\int_{0}^{\infty }P_{r}(T_{i}>t)=\sum_{j}m_{ij}$$ (2)

where $m_{ij}=-\frac{d}{ds}{\left[Q_{ij}^{**}(s)\right]}_{s=0}$.

Using Eq (2), mean sojourn time at state *S*_0_ is evaluated as follows:

$$\mu_{0}=\int_{0}^{\infty }\bar{F_{1}(t)}\bar{F_{2}(t)}\bar{F_{3}(t)}\bar{F_{4}(t)}\bar{F_{5}(t)}dt$$ (3)

Taking Laplace transform on Eq (3) both side, we get

$$\mu_{0}^{**}(s)=\int_{0}^{\infty }e^{-\theta_{1}t^{\eta }}e^{-\theta_{2}t^{\eta }}e^{-\theta_{3}t^{\eta }}e^{-\theta_{4}t^{\eta }}e^{-\theta_{5}t^{\eta }}e^{-st}dt$$

After solving it, we get

$$\mu_{0}^{**}(s)=\underset{s\to 0}{\lim}\frac{\Gamma (1+1/\eta )}{{\left(\theta_{1}+\theta_{2}+\theta_{3}+\theta_{4}+\theta_{5}+s\right)}^{1/\eta }}⟹\mu_{0}=\frac{\Gamma (1+1/\eta )}{{\left(\theta_{1}+\theta_{2}+\theta_{3}+\theta_{4}+\theta_{5}\right)}^{1/\eta }}$$

Similarly

$$\mu_{1}=\frac{\Gamma (1+1/\eta )}{{\left(\theta_{1}+\theta_{2}+\theta_{3}+\theta_{4}+\theta_{5}+\beta_{5}\right)}^{1/\eta }},\mu_{2}=\frac{\Gamma (1+1/\eta )}{{\left(\beta_{1}\right)}^{1/\eta }},\mu_{3}=\frac{\Gamma (1+1/\eta )}{{\left(\beta_{2}\right)}^{1/\eta }},\mu_{4}=\frac{\Gamma (1+1/\eta )}{{\left(\beta_{3}\right)}^{1/\eta }},\mu_{5}=\frac{\Gamma (1+1/\eta )}{{\left(\beta_{4}\right)}^{1/\eta }}$$

### 3.3 Mean time to system failure

To evaluate turbogenerator reliability **R**_i_(t) at time “t” starting from regenerative state S_i_ to a failed state S_j_, it represents the c.d.f of first passage time. The detailed methodology of mean time of system failure evaluation is presented in [37]. By considering failed states as absorbing state and using probabilistic arguments, following recursive relations for R_i_(t) are derived based on state transition diagram given in Fig 2:

$$R_{0}(t)=Q_{01}(t)®R_{1}(t)+Z_{0}(t)$$ (4)

$$R_{1}(t)=Q_{10}(t)®R_{0}(t)+Z_{1}(t)$$ (5)

Where,

$$Z_{0}(t)=Q_{02}(t)+Q_{03}(t)+Q_{04}(t)+Q_{05}(t)$$ (6)

$$Z_{1}(t)=Q_{16}(t)+Q_{17}(t)+Q_{18}(t)+Q_{19}(t)+Q_{1,10}(t)$$ (7)

By taking Laplace transformation of Eqs (4–5) and solving for $R_{0}^{**}(s)$, we get

$$R_{0}^{**}(s)=\frac{N_{0}}{D_{0}}=\frac{Z_{0}^{**}(s)+Q_{01}^{**}(s)Z_{1}^{**}(s)}{1-Q_{01}^{**}(s)Q_{10}^{**}(s)}$$ (8)

The inverse Laplace transformation of Eq (8) gives the reliability of turbogenerator. the mean time to system failure is derived as follows:

$$MTSF=\underset{s\to 0}{\lim}\frac{1-R_{0}^{**}(s)}{s}=\underset{s\to 0}{\lim}\frac{1-Q_{01}^{**}(s)Q_{10}^{**}(s)-Z_{0}^{**}(s)-Q_{01}^{**}(s)Z_{1}^{**}(s)}{s(1-Q_{01}^{**}(s)Q_{10}^{**}(s))}=\frac{\mu_{0}+P_{01}\mu_{1}}{1-P_{01}P_{10}}=\frac{a[(\sum \theta_{i}+\theta_{5})(\sum \theta_{i}+\theta_{5}+\beta_{5})+\theta_{5}{\left(\sum \theta_{i}+\theta_{5}\right)}^{1/\eta }{\left(\sum \theta_{i}+\theta_{5}+\beta_{5}\right)}^{1-1/\eta }]}{\left[{\left(\sum \theta_{i}+\theta_{5}\right)}^{1+1/\eta }(\sum \theta_{i}+\theta_{5}+\beta_{5})-\theta_{5}\beta_{5}{\left(\sum \theta_{i}+\theta_{5}\right)}^{1/\eta }\right]}$$ (9)

where *a* = *Γ*(1+1/*η*)

### 3.4 Availability analysis

Let A_i_(t) be the probability of turbogenerator that it is in up-state at instant ‘t’ given that the system entered regenerative state S_i_ at t = 0. The recursive relations for A_i_(t) are derived based on state transition diagram given in Fig 2::

$$A_{0}(t)=Z_{0}(t)+Q_{01}(t)®A_{1}(t)+Q_{02}(t)®A_{2}(t)+Q_{03}(t)®A_{3}(t)+Q_{04}(t)®A_{4}(t)+Q_{05}(t)®A_{5}(t)$$ (10)

$$A_{1}(t)=Z_{1}(t)+Q_{10}(t)®A_{0}(t)+Q_{12}^{6}(t)®A_{2}(t)+Q_{13}^{7}(t)®A_{3}(t)+Q_{14}^{8}(t)®A_{4}(t)+Q_{15}^{9}(t)®A_{5}(t)+Q_{11}^{10}(t)®A_{1}(t)$$ (11)

$$A_{2}(t)=Q_{20}(t)®A_{0}(t)$$ (12)

$$A_{3}(t)=Q_{30}(t)®A_{0}(t)$$ (13)

$$A_{4}(t)=Q_{40}(t)®A_{0}(t)$$ (14)

$$A_{5}(t)=Q_{50}(t)®A_{0}(t)$$ (15)

Taking Laplace transformation on Eqs (10–15) and solving for $A_{0}^{**}(s)$ *we get*

$$A_{0}^{**}(s)=\frac{N_{1}(s)}{D_{1}(s)}$$ (16)

Where,

$$D_{1}=[1-Q_{1.10}^{**}(s)][A+CQ_{02}^{**}(s)+DQ_{03}^{**}(s)+EQ_{04}^{**}(s)+FQ_{05}^{**}(s)]+Q_{01}^{**}(s)[B+CQ_{16}^{**}(s)+DQ_{17}^{**}(s)+EQ_{18}^{**}(s)+FQ_{19}^{**}(s)]$$

$$N_{1}=Z_{0}^{**}(s)[1-Q_{1,10}^{**}(s)]+Q_{01}^{**}(s)Z_{1}^{**}(s)$$

$$A=1-Q_{01}^{**}(s)-Q_{02}^{**}(s)-Q_{03}^{**}(s)-Q_{04}^{**}(s)-Q_{05}^{**}(s)$$

$$B=1-Q_{11}^{10**}(s)-Q_{12}^{6**}(s)-Q_{13}^{7**}(s)-Q_{14}^{8**}(s)-Q_{15}^{9**}(s)-Q_{10}^{**}(s)$$

$$C=1-Q_{20}^{**}(s)$$

$$D=1-Q_{30}^{**}(s)$$

$$E=1-Q_{40}^{**}(s)$$

$$F=1-Q_{50}^{**}(s)$$

After taking inverse Laplace transformation Eq (16), we get

$$Availability=\underset{s\to 0}{\lim}A_{0}^{**}(s)=\underset{s\to 0}{\lim}\frac{N_{1}+sN\prime_{1}}{D\prime_{1}}$$

$$=\frac{\mu_{0}(1-P_{1,10})+P_{01}\mu_{1}}{\left[1-P_{1,10}][\mu_{0}+P_{02}m_{20}+P_{03}m_{30}+P_{04}m_{40}+P_{05}m_{50}]+P_{01}[\mu_{1}+P_{16}m_{20}+P_{17}m_{30}+P_{18}m_{40}+P_{19}m_{50}\right]}$$ (17)

### 3.5 Busy period of server

Let B_i_(t) be the probability that repairman is busy in repairing the failed unit at epoch “t‟ given that the turbogenerator system entered state S_i_ at t = 0. The recursive relations for B_i_(t) are derived based on state transition diagram given in Fig 2::

$$B_{0}(t)=Q_{01}(t)®B_{1}(t)+Q_{02}(t)®B_{2}(t)+Q_{03}(t)®B_{3}(t)$$ (18)

$$B_{1}(t)=Q_{10}(t)®B_{0}(t)+Q_{12}^{6}(t)®B_{2}(t)+Q_{13}^{7}(t)®B_{3}(t)+Q_{14}^{8}(t)®B_{4}(t)+Q_{15}^{9}(t)®B_{5}(t)+Q_{11}^{10}(t)®B_{1}(t)$$ (19)

$$B_{2}(t)=Z_{2}(t)+Q_{20}(t)®B_{0}(t)$$ (20)

$$B_{3}(t)=Z_{3}(t)+Q_{30}(t)®B_{0}(t)$$ (21)

$$B_{4}(t)=Z_{4}(t)+Q_{40}(t)®B_{0}(t)$$ (22)

$$B_{5}(t)=Z_{5}(t)+Q_{50}(t)®B_{0}(t)$$ (23)

Taking Laplace transformation on both sides of Eqs (18–23) and solving for $B_{0}^{**}(s),$ *we get*

$$B_{0}^{**}(s)=\frac{N_{2}(s)}{D_{1}(s)}$$

Where,

$$N_{2}=[1-Q_{1,10}^{**}(s)][Z_{2}^{**}(s)Q_{02}^{**}(s)+Z_{3}^{**}(s)Q_{03}^{**}(s)+Z_{4}^{**}(s)Q_{04}^{**}(s)+Z_{5}^{**}(s)Q_{05}^{**}(s)]+Q_{01}^{**}(s)[Z_{2}^{**}(s)Q_{16}^{**}(s)+Z_{3}^{**}(s)Q_{17}^{**}(s)+Z_{4}^{**}(s)Q_{18}^{**}(s)+Z_{5}^{**}(s)Q_{19}^{**}(s)]$$

The busy period in steady state is given by as follows:

$$Busy\;Period\;of\;server=\underset{s\to 0}{\lim}\;B_{0}^{**}(s)=\underset{s\to 0}{\lim}\frac{N_{2}+sN\prime_{2}}{D\prime_{2}}$$

$$=\frac{\left[1-P_{1,10}][P_{02}\mu_{2}+P_{03}\mu_{3}+P_{04}\mu_{4}+P_{05}\mu_{5}]+P_{01}[P_{16}\mu_{2}+P_{17}\mu_{3}+P_{18}\mu_{4}+P_{19}\mu_{5}\right]}{\left[1-P_{1,10}][\mu_{0}+P_{02}m_{20}+P_{03}m_{30}+P_{04}m_{40}+P_{05}m_{50}]+P_{01}[\mu_{1}+P_{16}m_{20}+P_{17}m_{30}+P_{18}m_{40}+P_{19}m_{50}\right]}$$ (24)

### 3.6 Profit function

The expected profit *P* incurred by the system in long run is

$$P=k_{0}\;Availability-k_{1}\;Busy\;period\;of\;server$$ (25)

Where *k*_0_: *revenue per unit time*; *k*_1_: *cost per unit time*

## 4. Estimation of reliability measures under classical and Bayesian setups

### 4.1 Classical estimation

Let us assume that the failure (*f*_*i*_(.); *i* = 1,2,3,4,5,6) and repair (*g*_*i*_(.); *i* = 1,2,3,4,5,6) rates of various components of turbogenerator followed Weibull distribution having common shape and different scale parameters. Where:

$$f_{i}(t)=\theta_{i}\eta t^{\eta -1};\;i=1,2,3,4,5,6$$

$$g_{i}(t)=\beta_{i}\eta t^{\eta -1};\;i=1,2,3,4,5,6$$

Here, *θ*_*i*_&*β*_*i*_ are scale parameters while common scale parameter is *η*. All these random variables are statistically independent. As the main aim of present study is to estimate the parameters and reliability measures of turbogenerator in classical and Bayesian inferential setups. So, here maximum likelihood (ML) estimation method is employed as a powerful tool of classical estimation. The maximum likelihood estimators (MLE) of all the parameters are estimated for all the parameters of random variables.

Suppose ten independent random samples of size *n*_*i*_ (i = 1,2,3…….,10) are drawn from Weibull distribution with failure rates (*f*_*i*_(.); *i* = 1,2,3,4,5,6) and repair rates (*g*_*i*_(.); *i* = 1,2,3,4,5,6) respectively.


$${\hat{Y}}_{1}=(y_{11},y_{12},\ldots \ldots .y_{1n_{1}}){\hat{Y}}_{2}=(y_{21},y_{22},\ldots \ldots .y_{2n_{2}})$$

$${\hat{Y}}_{3}=(y_{31},y_{32},\ldots \ldots .y_{3n_{3}}){\hat{Y}}_{4}=(y_{41},y_{42},\ldots \ldots .y_{4n_{4}})$$

$${\hat{Y}}_{5}=(y_{51},y_{52},\ldots \ldots .y_{5n_{5}}){\hat{Y}}_{6}=(y_{61},y_{62},\ldots \ldots .y_{6n_{6}})$$

$${\hat{Y}}_{7}=(y_{71},y_{72},\ldots \ldots .y_{7n_{7}}){\hat{Y}}_{8}=(y_{81},y_{82},\ldots \ldots .y_{8n_{8}})$$

$${\hat{Y}}_{9}=(y_{91},y_{92},\ldots \ldots .y_{9n_{9}}){\hat{Y}}_{10}=(y_{10,1},y_{10,2},\ldots \ldots .y_{10,n_{10}})$$

The joint likelihood function based on above ten samples is given by

$$L=L({\hat{Y}}_{1},{\hat{Y}}_{2},{\hat{Y}}_{3},{\hat{Y}}_{4},{\hat{Y}}_{5},{\hat{Y}}_{6},{\hat{Y}}_{7},{\hat{Y}}_{8},{\hat{Y}}_{9},{\hat{Y}}_{10}|\theta_{1},\theta_{2},\theta_{3},\theta_{4},\theta_{5},\beta_{1},\beta_{2},\beta_{3},\beta_{4},\beta_{5})$$

$$L=\theta_{1}^{n_{1}}\theta_{2}^{n_{2}}\theta_{3}^{n_{3}}\theta_{4}^{n_{4}}\theta_{5}^{n_{5}}\beta_{1}^{n_{6}}\beta_{2}^{n_{7}}\beta_{3}^{n_{8}}\beta_{4}^{n_{9}}\beta_{5}^{n_{10}}\eta^{n_{1}+n_{2}+n_{3}+n_{4}+n_{5}+n_{6}+n_{7}+n_{8}+n_{9}+n_{10}}S_{1}S_{2}S_{3}S_{4}S_{5}S_{6}S_{7}S_{8}S_{9}S_{10}.e^{-(\theta_{1}T_{1}+\theta_{2}T_{2}+\theta_{3}T_{3}+\theta_{4}T_{4}+\theta_{5}T_{5}+\beta_{1}T_{6}+\beta_{2}T_{7}+\beta_{3}T_{8}+\beta_{4}T_{9}+\beta_{5}T_{10})}$$ (26)

where

$S_{i}=\prod_{j=1}^{n_{i}}y_{ij}^{\eta -1}$ and $T_{i}=\sum_{j=1}^{n_{i}}y_{ij}^{\eta }$ i = 1, 2,…….,10

Taking log on both side of Eq (26), we get

$$logL=n_{1}log\theta_{1}+\cdots \ldots .+n_{10}log\beta_{5}+\sum n_{i}log\eta +logS_{1}+\cdots \ldots .+logS_{10}-(\theta_{1}T_{1}+\ldots \ldots .+\beta_{5}T_{10})$$ (27)

The ML estimates (say ${\hat{\theta }}_{1},{\hat{\theta }}_{2},{\hat{\theta }}_{3},{\hat{\theta }}_{4},{\hat{\theta }}_{5},{\hat{\beta }}_{1},{\hat{\beta }}_{2},{\hat{\beta }}_{3},{\hat{\beta }}_{4},{\hat{\beta }}_{5},$) of the shape and scale parameters *θ*_1_, *θ*_2_, *θ*_3_, *θ*_4_, *θ*_5_, *β*_1_, *β*_2_, *β*_3_, *β*_4_, *β*_5_.


$${\hat{\theta }}_{1}=\frac{n_{1}}{\sum_{j=1}^{n_{1}}y_{ij}^{\eta }};\;{\hat{\theta }}_{2}=\frac{n_{2}}{\sum_{j=1}^{n_{2}}y_{ij}^{\eta }};\;{\hat{\theta }}_{3}=\frac{n_{3}}{\sum_{j=1}^{n_{3}}y_{ij}^{\eta }};\;{\hat{\theta }}_{4}=\frac{n_{4}}{\sum_{j=1}^{n_{4}}y_{ij}^{\eta }};\;\;{\hat{\theta }}_{5}=\frac{n_{5}}{\sum_{j=1}^{n_{5}}y_{ij}^{\eta }};\;{\hat{\beta }}_{1}=\frac{n_{6}}{\sum_{j=1}^{n_{6}}y_{ij}^{\eta }};\;{\hat{\beta }}_{2}=\frac{n_{7}}{\sum_{j=1}^{n_{7}}y_{ij}^{\eta }};$$

$${\hat{\beta }}_{3}=\frac{n_{8}}{\sum_{j=1}^{n_{8}}y_{ij}^{\eta }};\;{\hat{\beta }}_{4}=\frac{n_{9}}{\sum_{j=1}^{n_{9}}y_{ij}^{\eta }};\;{\hat{\beta }}_{5}=\frac{n_{10}}{\sum_{j=1}^{n_{10}}y_{ij}^{\eta }}$$ (28)

By using invariance property of invariance property of MLE, the expressions for MLE of MTSF, availability and profit function can be easily derived. Here $\hat{MTSF,}\;\hat{AV}$ and $\hat{P}$ represented the MLE of MTSF, availability and profit function respectively. The asymptotic distribution of

$${\left({\hat{\theta }}_{1}-\theta_{1},{\hat{\theta }}_{2}-\theta_{2},{\hat{\theta }}_{3}-\theta_{3},{\hat{\theta }}_{4}-\theta_{4},\ldots \ldots \ldots ..,{\hat{\beta }}_{5}-\beta_{5},\right)}^{\prime }∼N_{10}(0,I^{-1})$$

Here, I^-1^ represented the Fisher information matrix having diagonal elements

$$I_{11}=\frac{n_{1}}{\theta_{1}^{2}},I_{22}=\frac{n_{2}}{\theta_{2}^{2}},I_{33}=\frac{n_{3}}{\theta_{3}^{2}},I_{44}=\frac{n_{4}}{\theta_{4}^{2}},I_{55}=\frac{n_{5}}{\theta_{5}^{2}},I_{66}=\frac{n_{6}}{\beta_{1}^{2}},I_{77}=\frac{n_{7}}{\beta_{2}^{2}},I_{88}=\frac{n_{8}}{\beta_{3}^{2}},I_{99}=\frac{n_{9}}{\beta_{4}^{2}},I_{10,10}=\frac{n_{10}}{\beta_{5}^{2}}$$

And rest of the elements are equal to zero.

The asymptotic distribution of MTSF, availability and profit are as follows:

$$\left(\hat{MTSF}-MTSF)∼N_{10}(0,A^{\prime I^{-1}}A);(\hat{Av}-Av)∼N_{10}(0,B^{\prime I^{-1}}B);(\hat{P}-P)∼N_{10}(0,C^{\prime I^{-1}}C\right)$$

Where, $A^{\prime }={\left(\frac{\partial MTSF}{\partial \theta_{1}},\frac{\partial MTSF}{\partial \theta_{2}},\frac{\partial MTSF}{\partial \theta_{3}},\frac{\partial MTSF}{\partial \theta_{4}},\frac{\partial MTSF}{\partial \theta_{5}},\frac{\partial MTSF}{\partial \beta_{1}},\frac{\partial MTSF}{\partial \beta_{2}},\frac{\partial MTSF}{\partial \beta_{3}},\frac{\partial MTSF}{\partial \beta_{4}},\frac{\partial MTSF}{\partial \beta_{5}}\right)}^{\prime }$

$$B^{\prime }={\left(\frac{\partial AV}{\partial \theta_{1}},\frac{\partial AV}{\partial \theta_{2}},\frac{\partial AV}{\partial \theta_{3}},\frac{\partial AV}{\partial \theta_{4}},\frac{\partial AV}{\partial \theta_{5}},\frac{\partial AV}{\partial \beta_{1}},\frac{\partial AV}{\partial \beta_{2}},\frac{\partial AV}{\partial \beta_{3}},\frac{\partial AV}{\partial \beta_{4}},\frac{\partial AV}{\partial \beta_{5}}\right)}^{\prime }$$

$$C^{\prime }={\left(\frac{\partial P}{\partial \theta_{1}},\frac{\partial P}{\partial \theta_{2}},\frac{\partial P}{\partial \theta_{3}},\frac{\partial P}{\partial \theta_{4}},\frac{\partial P}{\partial \theta_{5}},\frac{\partial P}{\partial \beta_{1}},\frac{\partial P}{\partial \beta_{2}},\frac{\partial P}{\partial \beta_{3}},\frac{\partial P}{\partial \beta_{4}},\frac{\partial P}{\partial \beta_{5}}\right)}^{\prime }$$

Few of the expressions are shown in Appendix A (S1 Appendix).

### 4.2 Bayesian estimation

Bayesian estimation of parameters as well as reliability measures of turbogenerator is performed as it is considered that all parameters associated with failure and repair rates followed some distribution. In present study, all random variables followed two parameter Weibull distribution having known shape parameter (*η*). The family of gamma distributions is amply flexible as it can model a variety of prior information. Moreso, non-informative priors are particular cases of gamma priors. Also, the parameters of the gamma priors can be merged with model parameters, so that mathematical computations become easy. The scale parameter (θ_1_, θ_2_, θ_3_, θ_4_, θ_5_, β_1_, β_2_, β_3_, β_4_, β_5_) of distribution associate with random variables followed the Gamma distribution having parameters (termed as hyper parameters) (*α*_*i*_, *δ*_*i*_; *i* = 1,2,3,…,10) and described as given below:

$$\theta_{1}∼GAMMA(\alpha_{1},\delta_{1})$$

$$\theta_{2}∼GAMMA(\alpha_{2},\delta_{2})$$

$$\theta_{3}∼GAMMA(\alpha_{3},\delta_{3})$$

$$\theta_{4}∼GAMMA(\alpha_{4},\delta_{4})$$

$$\theta_{5}∼GAMMA(\alpha_{5},\delta_{5})$$

$$\beta_{1}∼GAMMA(\alpha_{6},\delta_{6})$$

$$\beta_{2}∼GAMMA(\alpha_{7},\delta_{7})$$

$$\beta_{3}∼GAMMA(\alpha_{8},\delta_{8})$$

$$\beta_{4}∼GAMMA(\alpha_{9},\delta_{9})$$

$$\beta_{5}∼GAMMA(\alpha_{10},\delta_{10})$$

The values of hyperparameters in the case of informative priors are taken in such a way that the mean of the prior distribution comes out equal to the true value of the parameter. The posterior distributions are derived using likelihood function (26) and prior distributions of θ_1_, θ_2_, θ_3_, θ_4_, θ_5_, β_1_, β_2_, β_3_, β_4_, β_5_ as follows:

$$\theta_{1}|{\underline{Y}}_{1}∼GAMMA(n_{1}+\alpha_{1},\delta_{1}+\sum_{j=1}^{n_{1}}y_{1j}^{\eta })$$ (29)

$$\theta_{2}|{\underline{Y}}_{2}∼GAMMA(n_{2}+\alpha_{2},\delta_{2}+\sum_{j=1}^{n_{2}}y_{2j}^{\eta })$$ (30)

$$\theta_{3}|{\underline{Y}}_{3}∼GAMMA(n_{3}+\alpha_{3},\delta_{3}+\sum_{j=1}^{n_{3}}y_{3j}^{\eta })$$ (31)

$$\theta_{4}|{\underline{Y}}_{4}∼GAMMA(n_{4}+\alpha_{4},\delta_{4}+\sum_{j=1}^{n_{4}}y_{4j}^{\eta })$$ (32)

$$\theta_{5}|{\underline{Y}}_{5}∼GAMMA(n_{5}+\alpha_{5},\delta_{5}+\sum_{j=1}^{n_{5}}y_{5j}^{\eta })$$ (33)

$$\beta_{1}|{\underline{Y}}_{6}∼GAMMA(n_{6}+\alpha_{6},\delta_{6}+\sum_{j=1}^{n_{6}}y_{6j}^{\eta })$$ (34)

$$\beta_{2}|{\underline{Y}}_{7}∼GAMMA(n_{7}+\alpha_{7},\delta_{7}+\sum_{j=1}^{n_{7}}y_{7j}^{\eta })$$ (35)

$$\beta_{3}|{\underline{Y}}_{8}∼GAMMA(n_{8}+\alpha_{8},\delta_{8}+\sum_{j=1}^{n_{8}}y_{9j}^{\eta })$$ (36)

$$\beta_{4}|{\underline{Y}}_{9}∼GAMMA(n_{9}+\alpha_{9},\delta_{9}+\sum_{j=1}^{n_{9}}y_{9j}^{\eta })$$ (37)

$$\beta_{10}|{\underline{Y}}_{10}∼GAMMA(n_{10}+\alpha_{10},\delta_{10}+\sum_{j=1}^{n_{10}}y_{10j}^{\eta })$$ (38)

The Bayes estimator of the scale parameters θ_1_, θ_2_, θ_3_, θ_4_, θ_5_, β_1_, β_2_, β_3_, β_4_, β_5_ under squared error loss function are the means of posterior distribution given in Eqs (29)–(38) and as follows:

$${\hat{\theta }}_{1}=\frac{\delta_{1}+\sum_{j=1}^{n_{1}}y_{1j}^{\eta }}{n_{1}+\theta_{1}}{\hat{\theta }}_{2}=\frac{\delta_{2}+\sum_{j=1}^{n_{2}}y_{2j}^{\eta }}{n_{2}+\theta_{2}}{\hat{\theta }}_{3}=\frac{\delta_{3}+\sum_{j=1}^{n_{3}}y_{3j}^{\eta }}{n_{3}+\theta_{3}}$$

$${\hat{\theta }}_{4}=\frac{\delta_{4}+\sum_{j=1}^{n_{4}}y_{4j}^{\eta }}{n_{4}+\theta_{4}}{\hat{\theta }}_{5}=\frac{\delta_{5}+\sum_{j=1}^{n_{5}}y_{5j}^{\eta }}{n_{5}+\theta_{5}}{\hat{\beta }}_{1}=\frac{\delta_{6}+\sum_{j=1}^{n_{6}}y_{6j}^{\eta }}{n_{6}+\beta_{1}}$$

$${\hat{\beta }}_{2}=\frac{\delta_{7}+\sum_{j=1}^{n_{7}}y_{7j}^{\eta }}{n_{7}+\beta_{2}}{\hat{\beta }}_{3}=\frac{\delta_{8}+\sum_{j=1}^{n_{8}}y_{8j}^{\eta }}{n_{8}+\beta_{3}},\;{\hat{\beta }}_{5}=\frac{\delta_{10}+\sum_{j=1}^{n_{10}}y_{10j}^{\eta }}{n_{10}+\beta_{5}}{\hat{\beta }}_{4}=\frac{\delta_{9}+\sum_{j=1}^{n_{9}}y_{9j}^{\eta }}{n_{9}+\beta_{4}}$$

## 5. Simulation study

In this section, MLE and Bayes estimates for parameters of Weibull distribution associated with failure and repair rates of turbogenerator are obtained. The MLE and Bayes estimates of scale parameters θ_1_, θ_2_, θ_3_, θ_4_, θ_5_, β_1_, β_2_, β_3_, β_4_, β_5_ and hence, by invariance property, for MTSF, availability and profit function are estimated under the assumption of known scale parameter. The theoretical results are validated through a simulation study. The comparison is made by using mean square error of estimates and width of confidence intervals. As the hazard rate of Weibull distribution is increasing, decreasing and constant according to the shape value of the parameter so investigation is also made for different values of shape parameters. Random sample of size 50 has been generated from Weibull distribution having various values of the parameters. The samples are generated for following set of values:

For η = 0.50, 1, and 2

- n = 50, θ1 = 0.01, θ2 = 0.05, θ3 = 0.06, θ4 = 0.065, θ5 = 0.045, β1 = 0.3, β2 = 0.4, β3 = 0.5, β4 = 0.6, β5 = 0.7
- n = 50, θ1 = 0.02, θ2 = 0.05, θ3 = 0.06, θ4 = 0.065, θ5 = 0.045, β1 = 0.3, β2 = 0.4, β3 = 0.5, β4 = 0.6, β5 = 0.7
- n = 50, θ1 = 0.03, θ2 = 0.05, θ3 = 0.06, θ4 = 0.065, θ5 = 0.045, β1 = 0.3, β2 = 0.4, β3 = 0.5, β4 = 0.6, β5 = 0.7
- n = 50, θ1 = 0.04, θ2 = 0.05, θ3 = 0.06, θ4 = 0.065, θ5 = 0.045, β1 = 0.3, β2 = 0.4, β3 = 0.5, β4 = 0.6, β5 = 0.7
- n = 50, θ1 = 0.05, θ2 = 0.05, θ3 = 0.06, θ4 = 0.065, θ5 = 0.045, β1 = 0.3, β2 = 0.4, β3 = 0.5, β4 = 0.6, β5 = 0.7
- n = 50, θ1 = 0.06, θ2 = 0.05, θ3 = 0.06, θ4 = 0.065, θ5 = 0.045, β1 = 0.3, β2 = 0.4, β3 = 0.5, β4 = 0.6, β5 = 0.7
- n = 50, θ1 = 0.07, θ2 = 0.05, θ3 = 0.06, θ4 = 0.065, θ5 = 0.045, β1 = 0.3, β2 = 0.4, β3 = 0.5, β4 = 0.6, β5 = 0.7
- n = 50, θ1 = 0.08, θ2 = 0.05, θ3 = 0.06, θ4 = 0.065, θ5 = 0.045, β1 = 0.3, β2 = 0.4, β3 = 0.5, β4 = 0.6, β5 = 0.7
- n = 50, θ1 = 0.09, θ2 = 0.05, θ3 = 0.06, θ4 = 0.065, θ5 = 0.045, β1 = 0.3, β2 = 0.4, β3 = 0.5, β4 = 0.6, β5 = 0.7
- n = 50, θ1 = 0.1, θ2 = 0.05, θ3 = 0.06, θ4 = 0.065, θ5 = 0.045, β1 = 0.3, β2 = 0.4, β3 = 0.5, β4 = 0.6, β5 = 0.7

**Fig 3:**
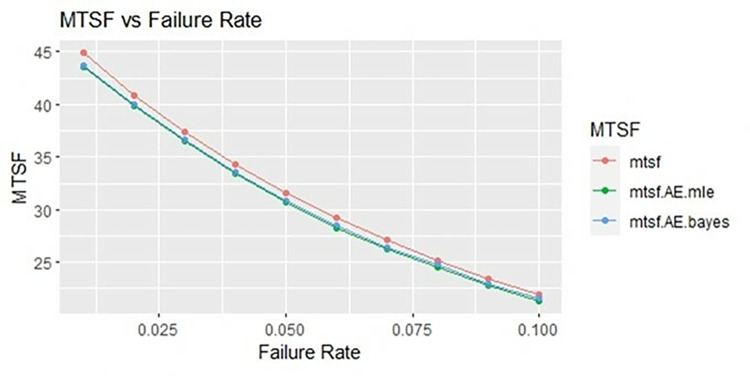
Behaviour of MTSF with varying failure rate (Ө_1_) for η = 0.5.

**Fig 4:**
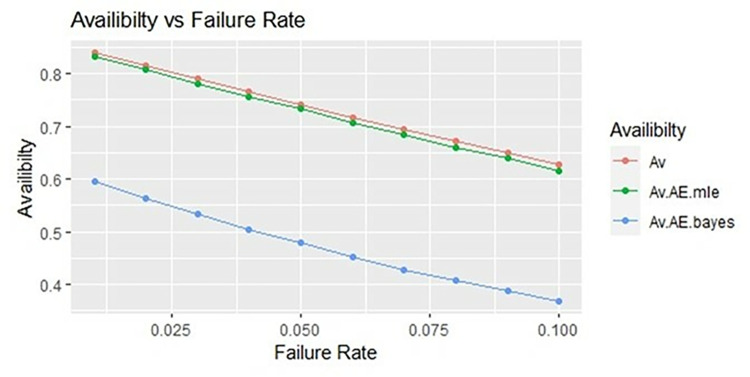
Behaviour of availability with varying failure rate (Ө_1_) for η = 0.5.

**Fig 5:**
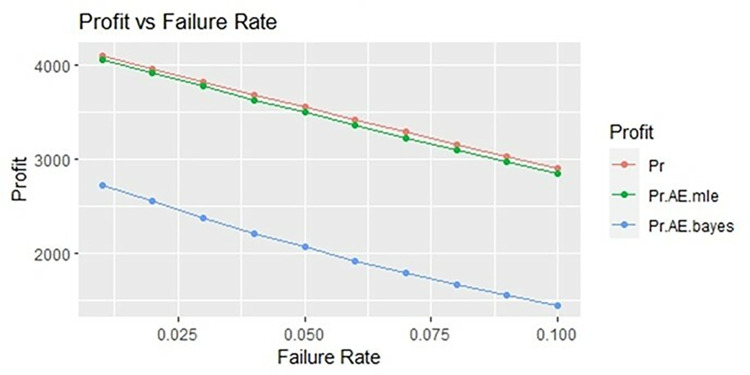
Behaviour of profit with varying failure rate (Ө_1_) for η = 0.5.

**Fig 6:**
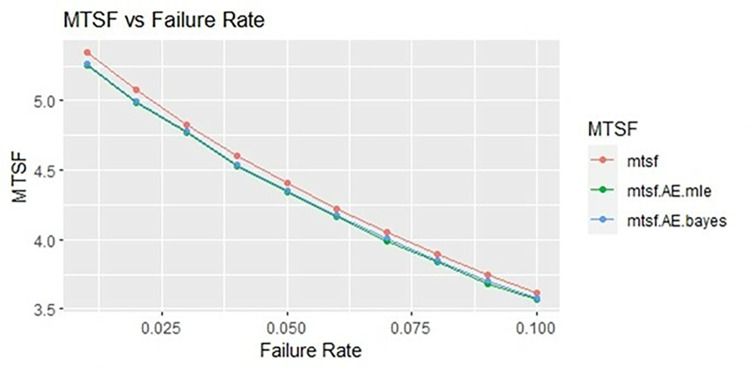
Behaviour of MTSF with varying failure rate (Ө_1_) for η = 1.

**Fig 7:**
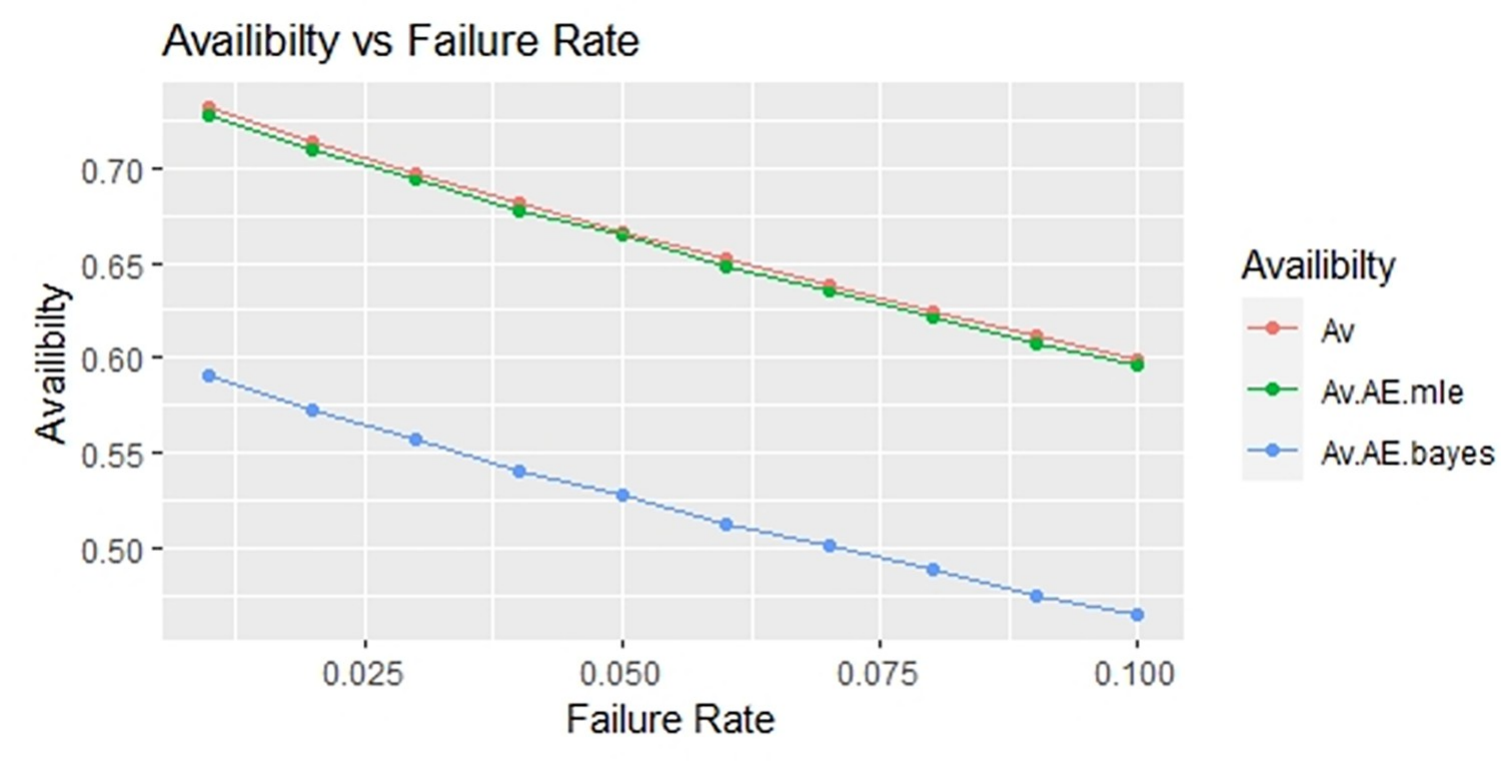
Behaviour of availability with varying failure rate (Ө_1_) for η = 1.

**Fig 8:**
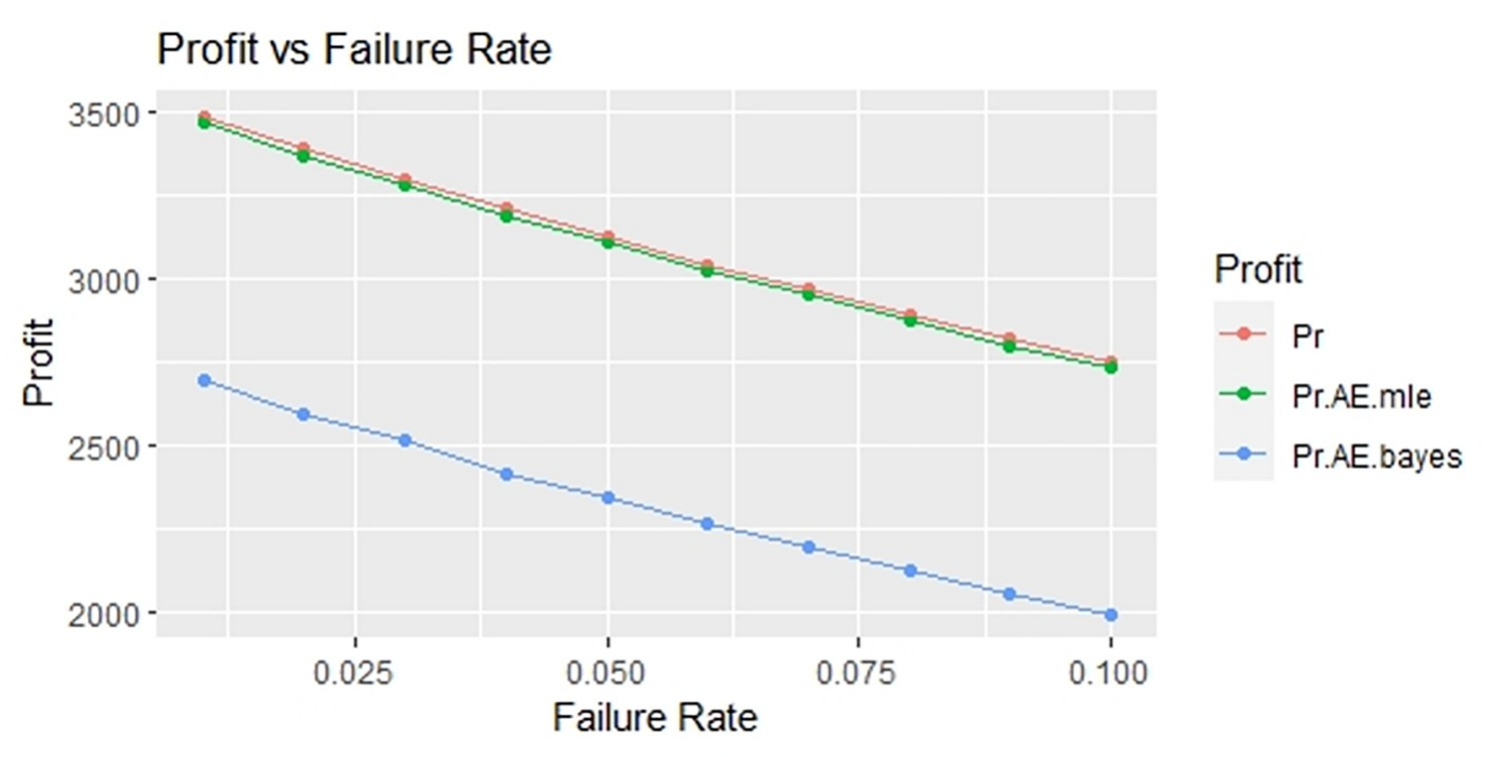
Behaviour of profit with varying failure rate (Ө_1_) for η = 1.

**Fig 9:**
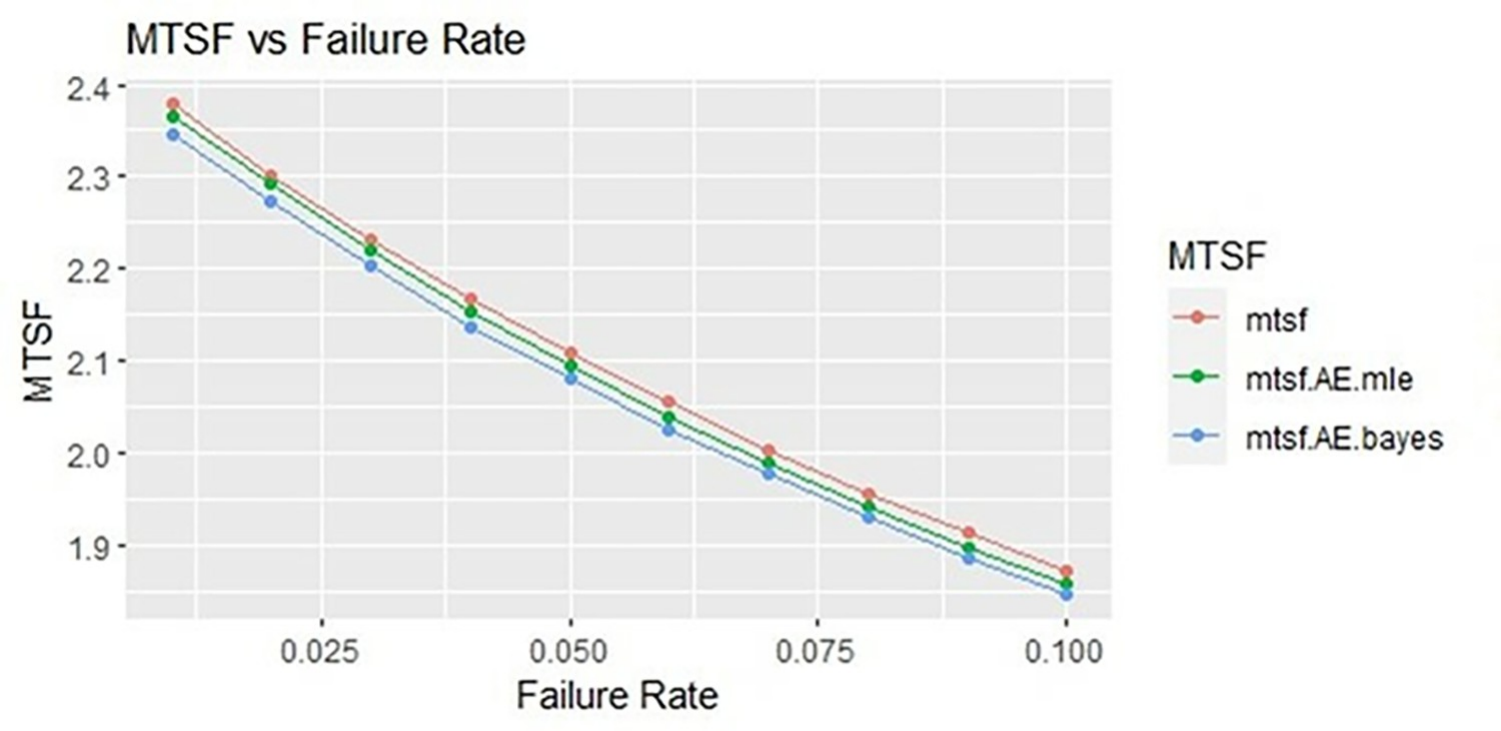
Behaviour of MTSF with varying failure rate (Ө_1_) for η = 2.

**Fig 10:**
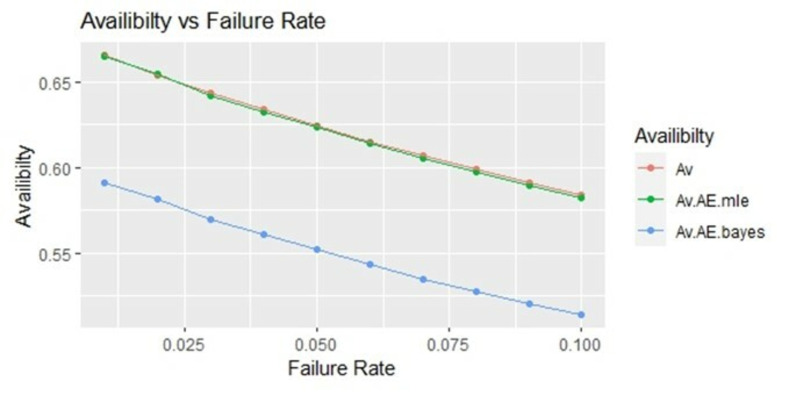
Behaviour of availability with varying failure rate (Ө_1_) for η = 2.

**Fig 11:**
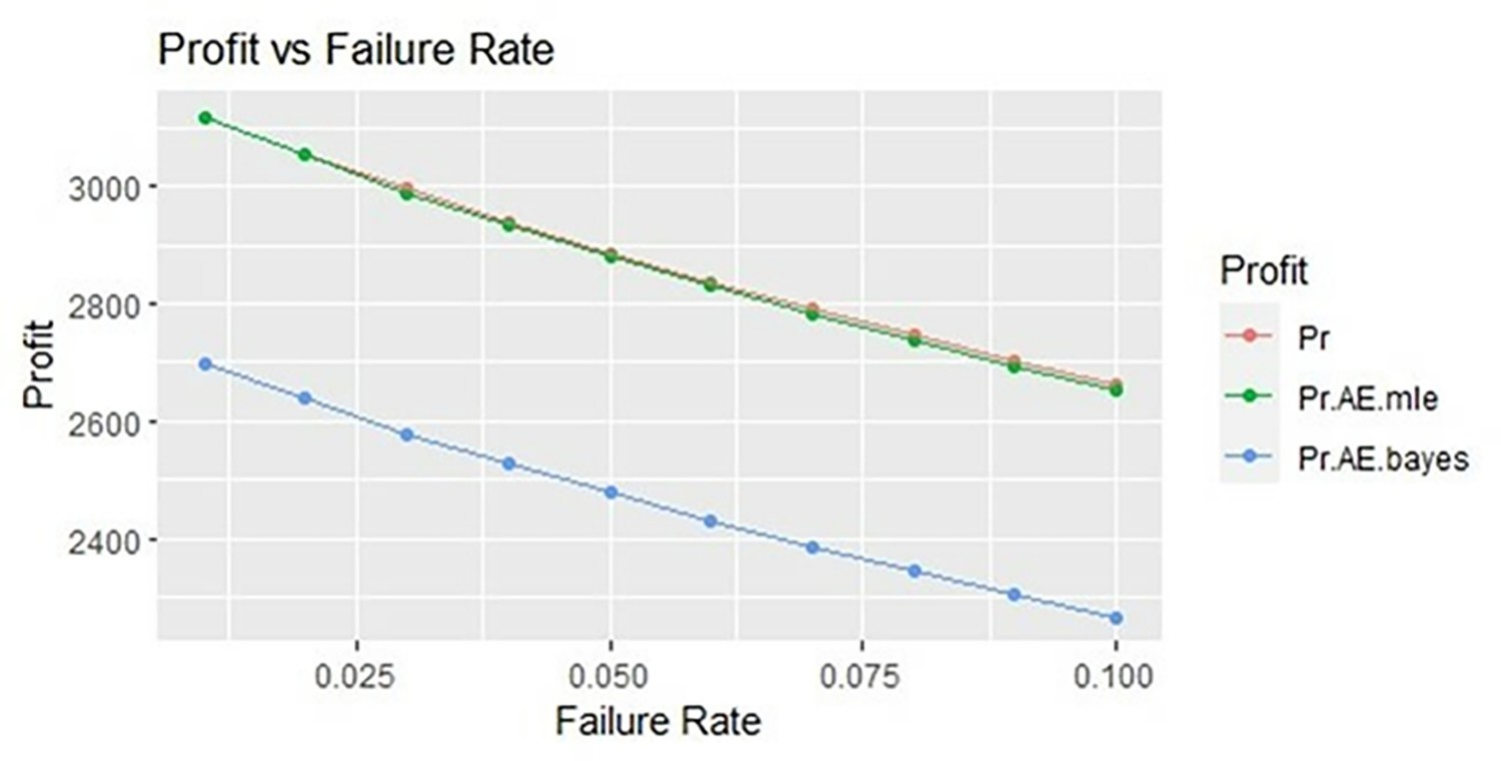
Behaviour of profit with varying failure rate (Ө_1_) for η = 2.

By using above values of parameters fifty random samples generated and MLE and Bayes estimated (for non-informative prior) of parameters, MTSF, availability and profit function is obtained. For Bayesian investigation 10000 realization by using non-informative prior and posterior densities. The values of the Gamma hyper parameters are obtained by setting ${\alpha /\beta }_{i}=\frac{b_{i}}{a_{i}}$. All the estimates along with true value, mean square errors, and length of intervals/HPD are summarized in Tables 1–7 and shown graphically in Figs 3–11. The profit function is evaluated by taking 5000 and 600, respectively. For all the numerical computations, the programs are developed in R-environment.

**Table 1:** Values of MTSF for fixed η = 0.5 and varying Ө_1_.

Estimates Ө_1_	0.1	0.2	0.3	0.4	0.5	0.6	0.7	0.8	0.9	1
True MTSF	44.8669	40.8505	37.3531	34.2887	31.5884	29.1965	27.0677	25.1645	23.4561	21.9167
MTSF_MLE_MSE	42.2584	30.5923	26.0558	20.6032	17.1161	15.2012	12.5612	10.2235	10.8187	9.1544
MTSF_MLE	43.549	39.8395	36.551	33.408	30.7116	28.2843	26.2396	24.5585	22.7489	21.3127
MTSF_Bayes	43.6571	39.9295	36.651	33.5432	30.8731	28.4728	26.4473	24.7617	22.9735	21.534
MTSF_Bayes_MSE	33.0806	24.1934	20.5569	16.3265	13.4604	11.8243	9.6613	7.7434	8.0673	6.7001
MTSF.length.MLE	24.6241	21.761	19.5038	17.5681	16.0427	14.7706	13.7609	12.9778	12.1461	11.5105
MTSF.length.Bayes	26.2679	23.2484	20.8689	18.8203	17.1967	15.8354	14.7239	13.8438	12.9215	12.1935

**Table 2:** Values of availability for fixed η = 0.5 and varying Ө_1_.

Estimates Ө_1_	0.1	0.2	0.3	0.4	0.5	0.6	0.7	0.8	0.9	1
True Availability	0.8386	0.8142	0.7897	0.7653	0.7411	0.7173	0.6938	0.6709	0.6484	0.6266
Avail_MLE_MSE	0.0009	0.0011	0.0012	0.0015	0.0018	0.002	0.0025	0.0026	0.0031	0.0032
Avail _MLE	0.8312	0.8064	0.7815	0.7546	0.7326	0.7075	0.6829	0.6594	0.6392	0.6161
Avail _Bayes	0.5945	0.564	0.5331	0.5027	0.4783	0.4521	0.4282	0.4069	0.3871	0.3677
Avail _Bayes_MSE	0.0611	0.064	0.0673	0.0704	0.0705	0.0717	0.072	0.071	0.0698	0.0684
Avail.length.MLE	0.1176	0.1258	0.1368	0.1504	0.1624	0.1758	0.1889	0.2017	0.2112	0.2218
Avail.length.Bayes	0.2162	0.2121	0.2114	0.2124	0.2138	0.2152	0.2163	0.2177	0.217	0.2167

**Table 3:** Values of profit for fixed η = 0.5 and varying Ө_1_.

Estimates Ө_1_	0.1	0.2	0.3	0.4	0.5	0.6	0.7	0.8	0.9	1
True Profit	4091.79	3954.65	3817.42	3680.82	3545.50	3411.99	3280.75	3152.17	3026.56	2904.16
Profit_MLE_MSE	28003.6	34026.4	38853.2	46939.5	55096.9	64020.4	77202.6	82288.9	96314.4	101361
Profit _MLE	4049.92	3910.75	3771.59	3620.97	3497.73	3356.58	3219.23	3087.30	2974.50	2844.87
Profit _Bayes	2717.86	2546.31	2373.97	2204.32	2067.07	1920.46	1787.27	1668.21	1557.02	1448.93
Profit_Bayes_MSE	1935187	2026877	2128307	2225109	2230367	2266927	2275512	2243309	2204649	2160122
Profit.length.MLE	656.17	702.42	764.47	840.24	907.55	983.04	1055.79	1127.93	1180.64	1240.44
Profit.length.Bayes	1204.97	1182.48	1178.83	1184.22	1192.25	1200.32	1206.22	1213.67	1209.74	1208.04

**Table 4:** Values of MTSF for fixed η = 1 and varying Ө_1_.

Estimates Ө_1_	0.1	0.2	0.3	0.4	0.5	0.6	0.7	0.8	0.9	1
True MTSF	5.3454	5.0747	4.8301	4.608	4.4054	4.2198	4.0493	3.892	3.7464	3.6114
MTSF_MLE_MSE	0.1703	0.1537	0.1307	0.114	0.0962	0.0906	0.0838	0.0831	0.0725	0.0657
MTSF_MLE	5.2575	4.9898	4.7724	4.5297	4.338	4.1662	3.9914	3.8367	3.6827	3.5676
MTSF_Bayes	5.2629	4.9958	4.7774	4.5379	4.3472	4.1749	4.0027	3.8493	3.698	3.5808
MTSF_Bayes_MSE	0.1331	0.1207	0.1028	0.0897	0.0755	0.0706	0.0643	0.0629	0.0536	0.0475
MTSF.length.MLE	1.6001	1.463	1.3632	1.2724	1.2091	1.1581	1.1136	1.0781	1.0457	1.023
MTSF.length.Bayes	1.7055	1.562	1.4571	1.361	1.293	1.2368	1.1875	1.1463	1.1076	1.0779

**Table 5:** Values of availability for fixed η = 1 and varying Ө_1_.

Estimates Ө_1_	0.1	0.2	0.3	0.4	0.5	0.6	0.7	0.8	0.9	1
True Availability	0.7312	0.7138	0.6972	0.6814	0.6663	0.6518	0.6379	0.6246	0.6119	0.5997
Avail_MLE_MSE	0.00060	0.00060	0.00060	0.00060	0.00060	0.00060	0.00070	0.00080	0.00090	0.00080
Avail _MLE	0.7282	0.7097	0.6947	0.6772	0.6643	0.6484	0.6358	0.622	0.6076	0.5968
Avail _Bayes	0.5901	0.5724	0.5577	0.5406	0.5277	0.5129	0.5005	0.488	0.4749	0.4647
Avail _Bayes_MSE	0.0205	0.0205	0.02	0.0203	0.0197	0.0198	0.0194	0.0192	0.0193	0.0187
Avail.length.MLE	0.0949	0.0937	0.0936	0.0956	0.0976	0.1006	0.1037	0.1071	0.1108	0.1134
Avail.length.Bayes	0.1241	0.12	0.1174	0.1167	0.1163	0.1168	0.1177	0.1188	0.12	0.121

**Table 6:** Values of profit for fixed η = 1 and varying Ө_1_.

Estimates Ө_1_	0.1	0.2	0.3	0.4	0.5	0.6	0.7	0.8	0.9	1
True Profit	3486.31	3389.11	3296.43	3207.95	3123.40	3042.53	2965.1	2890.87	2819.69	2751.34
Profit_MLE_MSE	17888.00	18296.53	18522.70	17716.20	18821.06	19629.77	21114.60	23305.48	26621.10	24743.46
Profit _MLE	3469.34	3365.84	3282.13	3184.82	3111.92	3023.25	2952.8	2875.63	2795.10	2734.93
Profit _Bayes	2692.22	2593.12	2511.06	2416.18	2343.16	2261.28	2191.9	2121.85	2049.03	1992.62
Profit_Bayes_MSE	646672.04	649351.23	632477.23	640665.58	622381.62	624522.96	612048.05	606316.80	608988.44	589007.17
Profit.length.MLE	517.32	511.25	511.86	523.69	535.72	553.25	571.4	591.11	612.53	627.50
Profit.length.Bayes	675.04	653.18	639.98	636.35	636.04	639.27	645.7	652.38	660.10	666.37

**Table 7:** Values of MTSF for fixed η = 2 and varying Ө_1_.

Estimates Ө_1_	0.1	0.2	0.3	0.4	0.5	0.6	0.7	0.8	0.9	1
True MTSF	2.3779	2.3018	2.2322	2.1682	2.1092	2.0545	2.0037	1.9564	1.9121	1.8706
MTSF_MLE_MSE	0.015	0.0125	0.0108	0.0106	0.0093	0.0085	0.0085	0.0083	0.0075	0.0072
MTSF_MLE	2.3658	2.2938	2.2213	2.1529	2.0945	2.0398	1.9896	1.9412	1.8968	1.8562
MTSF_Bayes	2.3461	2.2745	2.2036	2.137	2.0796	2.0261	1.9768	1.9295	1.8862	1.8462
MTSF_Bayes_MSE	0.012	0.0099	0.0089	0.0087	0.0077	0.007	0.0069	0.0066	0.0059	0.0056
MTSF.length.MLE	0.4751	0.4415	0.4134	0.3908	0.3745	0.3609	0.3507	0.342	0.3347	0.3296
MTSF.length.Bayes	0.4943	0.4598	0.4312	0.4083	0.3914	0.377	0.3658	0.3559	0.3473	0.3405

It is observed from numerical values given in Tables 1–3 that mean time to system failure, availability and profit incurred by turbogenerator decreases with the increase of failure rate Ө_1_. The MLE and Bayes estimates of MTSF, availability and profit of turbogenerator also exhibit the same pattern with respect to failure rate Ө_1_. The mean square error of MLE and Bayes estimators derived and found that it is less in maximum likelihood estimation along with confidence intervals length at η = 0.5. The same pattern is also shown graphically as mean time to system failure (Fig 3), availability (Fig 6) and profit (Fig 9).

For the shape parameter η = 1, it is revealed from numerical values given in Tables 4–6 that mean time to system failure, availability and profit incurred by turbogenerator decreases with the increase of failure rate Ө_1_. It is observed that true value, MLE and Bayes estimates of MTSF at Ө_1_ = 0.1 attained the values 5.3454, 5.2575 and 5.2629 respectively. The MLE and Bayes estimates of availability and profit of turbogenerator also exhibit the same pattern with respect to failure rate Ө_1_. The mean square error of MLE and Bayes estimators derived and found that it is less in maximum likelihood estimation along with confidence intervals length for η = 1. The same pattern is also shown graphically as mean time to system failure (Fig 4), availability (Fig 8) and profit (Fig 10).

For the shape parameter η = 2, it is revealed from numerical values given in Tables 7–9 that mean time to system failure, availability and profit incurred by turbogenerator decreases with the increase of failure rate Ө_1_. It is observed that true value, MLE and Bayes estimates of MTSF at Ө_1_ = 0.1 attained the values 2.3779, 2.3658 and 2.3461 respectively. The MLE and Bayes estimates of availability and profit of turbogenerator also exhibit the same pattern with respect to failure rate Ө_1_. The mean square error of MLE and Bayes estimators derived and found that it is less in maximum likelihood estimation along with confidence intervals length for η = 2. The same pattern is also shown graphically as mean time to system failure (Fig 5), availability (Fig 8) and profit (Fig 11). The numerical results exhibit that the numerical values of estimators declined with respect to the increase in shape parameter η = 0.5, 1 & 2 respectively.

**Table 8:** Values of availability for fixed η = 2 and varying Ө_1_.

Estimates Ө_1_	0.1	0.2	0.3	0.4	0.5	0.6	0.7	0.8	0.9	1
True Availability	0.6657	0.6544	0.6438	0.6338	0.6243	0.6154	0.607	0.5989	0.5913	0.584
Avail_MLE_MSE	0.00040	0.00040	0.00030	0.00030	0.00030	0.00030	0.00030	0.00030	0.00030	0.00030
Avail _MLE	0.6655	0.6546	0.6424	0.6329	0.6236	0.6142	0.6054	0.5974	0.5899	0.5826
Avail _Bayes	0.5914	0.5812	0.5698	0.5605	0.552	0.5431	0.5347	0.5274	0.5203	0.5137
Avail _Bayes_MSE	0.0058	0.0057	0.0057	0.0056	0.0055	0.0055	0.0055	0.0053	0.0053	0.0052
Avail.length.MLE	0.076	0.0729	0.0711	0.0697	0.0689	0.0688	0.0688	0.069	0.0694	0.0698
Avail.length.Bayes	0.0871	0.0833	0.0807	0.0786	0.0773	0.0764	0.0757	0.0754	0.0751	0.0749

**Table 9:** Values of profit for fixed η = 2 and varying Ө_1_.

Estimates Ө_1_	0.1	0.2	0.3	0.4	0.5	0.6	0.7	0.8	0.9	1
True Profit	3117.19	3054.22	2995.12	2939.50	2887.01	2837.36	2790.29	2745.57	2703.01	2662.42
Profit_MLE_MSE	10224.52	9689.77	8972.54	8979.80	8435.52	8396.81	9543.60	9188.36	9700.55	9297.67
Profit _MLE	3115.94	3054.99	2988.03	2933.98	2882.78	2829.86	2781.76	2737.15	2695.05	2654.65
Profit _Bayes	2698.68	2641.74	2579.43	2526.97	2479.77	2430.35	2384.26	2343.27	2303.30	2267.07
Profit_Bayes_MSE	183164.99	178114.87	179840.58	177046.83	171950.35	171708.86	171642.92	168017.89	166012.92	162168.78
Profit.length.MLE	397.14	381.54	372.61	366.02	363.55	363.68	365.17	367.91	371.11	374.82
Profit.length.Bayes	454.09	434.44	421.51	411.24	405.39	401.68	399.58	399.00	398.75	399.19

## 6. Conclusion

In present study, the classical and Bayesian estimation of the reliability characteristics is performed of a turbogenerator system. For a particular set of parametric values true MTSF, steady state availability and profit function are evaluated. Tables 1–9 reflected that MTSF, availability and profit decrease with the failure rate (θ1) of turbine governing unit. The values of mean time to system failure, availability and profit sharply declined with the increase of the shape parameter η = 0.5, 1 and 2. From the simulation results as shown in Tables 1–9, it is observed that for the shape parameter η = 0.5, 1 and 2 the true value of MTSF, availability, profit, MLE and Bayes estimates of MTSF, MLE and Bayes estimates of availability and MLE and Bayes estimates of profit decreases with respect to failure rate (θ1) of turbine governing unit. The mean square error (MSE) of maximum likelihood estimators and width of confidence intervals of MTSF, availability and profit are less in comparison of the Bayes MSE and HPD for η = 0.5, 1 and 2. Hence, it is recommended that to use ML estimated over Bayes estimation for estimation of reliability characteristics of turbogenerator. The work may be further extended by considering other informative priors for the distribution. Further, the proposed methodology may be opted for the reliability evaluation of other similar kind of mechanical systems as well as in process industries.

## Supporting information

S1 Appendix (DOCX)
