# Supplementary material for: Stochastic modeling and parameter estimation of turbogenerator unit of a thermal power plant under classical and Bayesian inferential framework
Source: PLoS One. 2023 Oct 20;18(10):e0292154. doi: 10.1371/journal.pone.0292154 (PMC10588864; doi:10.1371/journal.pone.0292154)
Supplement: S1 Appendix — (DOCX) [file pone.0292154.s001.docx]

**Appendix:**

1. Partial derivatives of MTSF, availability and profit can be derived as follows:

$$\begin{aligned} \frac{\partial MTSF}{\partial\theta_{1}}=\frac{a\left[ \left( \sum\theta_{i} \right)+\left( \sum\theta_{i}+\beta_{5} \right)+\left( \frac{1}{\eta} \right)\theta_{5}\left( \sum\theta_{i} \right)^{\frac{1}{\eta}-1}\left( \sum\theta_{i}+\beta_{5} \right)^{1-1/\eta}+\theta_{5}\left( \sum\theta_{i} \right)^{\frac{1}{\eta}}\left( 1-\frac{1}{\eta} \right)\left( \sum\theta_{i}+\beta_{5} \right)^{\frac{-1}{\eta}} \right]}{\left[ \left( \sum\theta_{i} \right)^{1+1/\eta}\left( \sum\theta_{i}+\beta_{5} \right)-\theta_{5}\beta_{5}\left( \sum\theta_{i} \right)^{1/\eta} \right]} \\ -\frac{a\left[ \left( \sum\theta_{i} \right)\left( \sum\theta_{i}+\beta_{5} \right)+\theta_{5}\left( \sum\theta_{i} \right)^{\frac{1}{\eta}}\left( \sum\theta_{i}+\beta_{5} \right)^{1-\frac{1}{\eta}} \right]\left[ \left( 1+\frac{1}{\eta} \right)\left( \sum\theta_{i} \right)^{\frac{1}{\eta}}\left( \sum\theta_{i}+\beta_{5} \right)+\left( \sum\theta_{i} \right)^{1+\frac{1}{\eta}}-\left( \frac{1}{\eta} \right)\theta_{5}\beta_{5}\left( \sum\theta_{i} \right)^{\frac{1}{\eta}-1} \right]}{\left[ \left( \sum\theta_{i} \right)^{1+\frac{1}{\eta}}\left( \sum\theta_{i}+\beta_{5} \right)-\theta_{5}\beta_{5}\left( \sum\theta_{i} \right)^{\frac{1}{\eta}} \right]^{2}} \end{aligned}$$

similarly

$$\frac{\partial MTSF}{\partial\theta_{1}}=\frac{\partial MTSF}{\partial\theta_{2}}=\frac{\partial MTSF}{\partial\theta_{3}}=\frac{\partial MTSF}{\partial\theta_{4}}$$

$$\begin{aligned} \frac{\partial MTSF}{\partial\theta_{5}}=a\frac{\left[ \left( \sum\theta_{i} \right)+\left( \sum\theta_{i}+\beta_{5} \right)+\left( \sum\theta_{i} \right)^{\frac{1}{\eta}}+\frac{1}{\eta}\theta_{5}\left( \sum\theta_{i} \right)^{\frac{1}{\eta}-1}\left( \sum\theta_{i}+\beta_{5} \right)^{1-1/\eta} \right]}{\left[ \left( \sum\theta_{i} \right)^{1+1/\eta}\left( \sum\theta_{i}+\beta_{5} \right)-\theta_{5}\beta_{5}\left( \sum\theta_{i} \right)^{1/\eta} \right]}\text{ } \\ \text{ }+\frac{a\left[ \theta_{5}\left( \sum\theta_{i} \right)^{\frac{1}{\eta}}\left( 1-\frac{1}{\eta} \right)\left( \sum\theta_{i}+\beta_{5} \right)^{\frac{-1}{\eta}} \right]}{\left[ \left( \sum\theta_{i} \right)^{1+1/\eta}\left( \sum\theta_{i}+\beta_{5} \right)-\theta_{5}\beta_{5}\left( \sum\theta_{i} \right)^{1/\eta} \right]} \\ \text{ }-\frac{a\left[ \left( \sum\theta_{i} \right)\left( \sum\theta_{i}+\beta_{5} \right)+\theta_{5}\left( \sum\theta_{i} \right)^{\frac{1}{\eta}}\left( \sum\theta_{i}+\beta_{5} \right)^{1-\frac{1}{\eta}} \right]\left[ \left( 1+\frac{1}{\eta} \right)\left( \sum\theta_{i} \right)^{\frac{1}{\eta}}\left( \sum\theta_{i}+\beta_{5} \right) \right]}{\left[ \left( \sum\theta_{i} \right)^{1+\frac{1}{\eta}}\left( \sum\theta_{i}+\beta_{5} \right)-\theta_{5}\beta_{5}\left( \sum\theta_{i} \right)^{\frac{1}{\eta}} \right]^{2}} \\ \text{ }-\frac{a\left[ \left( \sum\theta_{i} \right)\left( \sum\theta_{i}+\beta_{5} \right)+\theta_{5}\left( \sum\theta_{i} \right)^{\frac{1}{\eta}}\left( \sum\theta_{i}+\beta_{5} \right)^{1-\frac{1}{\eta}} \right]\left[ \left( \sum\theta_{i} \right)^{1+\frac{1}{\eta}}-\beta_{5}\left( \sum\theta_{i} \right)^{1/\eta}-\left( \frac{1}{\eta} \right)\theta_{5}\beta_{5}\left( \sum\theta_{i} \right)^{\frac{1}{\eta}-1} \right]}{\left[ \left( \sum\theta_{i} \right)^{1+\frac{1}{\eta}}\left( \sum\theta_{i}+\beta_{5} \right)-\theta_{5}\beta_{5}\left( \sum\theta_{i} \right)^{\frac{1}{\eta}} \right]^{2}} \end{aligned}$$

$$\begin{aligned} \frac{\partial MTSF}{\partial\beta_{5}}=\frac{a\left[ \left( \sum\theta_{i} \right)+\theta_{5}\left( \sum\theta_{i} \right)^{\frac{1}{\eta}}\left( 1-\frac{1}{\eta} \right)\left( \sum\theta_{i}+\beta_{5} \right)^{\frac{-1}{\eta}} \right]}{\left[ \left( \sum\theta_{i} \right)^{1+1/\eta}\left( \sum\theta_{i}+\beta_{5} \right)-\theta_{5}\beta_{5}\left( \sum\theta_{i} \right)^{1/\eta} \right]}\text{ } \\ \text{ }-\frac{a\left[ \left( \sum\theta_{i} \right)\left( \sum\theta_{i}+\beta_{5} \right)+\theta_{5}\left( \sum\theta_{i} \right)^{\frac{1}{\eta}}\left( \sum\theta_{i}+\beta_{5} \right)^{1-\frac{1}{\eta}} \right]\left[ \left( \sum\theta_{i} \right)^{1+\frac{1}{\eta}}-\theta_{5}\left( \sum\theta_{i} \right)^{\frac{1}{\eta}} \right]}{\left[ \left( \sum\theta_{i} \right)^{1+\frac{1}{\eta}}\left( \sum\theta_{i}+\beta_{5} \right)-\theta_{5}\beta_{5}\left( \sum\theta_{i} \right)^{\frac{1}{\eta}} \right]^{2}} \end{aligned}$$

$$Rest of the expression can be obtained in similar way.$$
